# Factors confounding the assessment of reflection: a critical review

**DOI:** 10.1186/1472-6920-11-104

**Published:** 2011-12-28

**Authors:** Sebastiaan Koole, Tim Dornan, Leen Aper, Albert Scherpbier, Martin Valcke, Janke Cohen-Schotanus, Anselme Derese

**Affiliations:** 1 Centre for Educational Development, Faculty of Medicine and Health Sciences, Ghent University, Ghent, Belgium; 2 Department of Educational Development and Research, Faculty of Health, Medicine and Life Sciences, Maastricht University, Maastricht, the Netherlands; 3 Institute for Medical Education, Faculty of Health, Medicine and Life Sciences, Maastricht University, the Netherlands; 4 Department of Educational Studies, Faculty of Psychology and Educational Sciences, Ghent University, Belgium; 5 Centre for Research and Innovation in Medical Education, Faculty of Medical Sciences, University of Groningen and University Medical Centre Groningen, Groningen, the Netherlands

## Abstract

**Background:**

Reflection on experience is an increasingly critical part of professional development and lifelong learning. There is, however, continuing uncertainty about how best to put principle into practice, particularly as regards assessment. This article explores those uncertainties in order to find practical ways of assessing reflection.

**Discussion:**

We critically review four problems: 1. Inconsistent definitions of reflection; 2. Lack of standards to determine (in)adequate reflection; 3. Factors that complicate assessment; 4. Internal and external contextual factors affecting the assessment of reflection.

**Summary:**

To address the problem of *inconsistency*, we identified processes that were common to a number of widely quoted theories and synthesised a model, which yielded six indicators that could be used in assessment instruments. We arrived at the conclusion that, until further progress has been made in *defining standards*, assessment must depend on developing and communicating local consensus between stakeholders (students, practitioners, teachers, supervisors, curriculum developers) about what is expected in exercises and formal tests. Major factors that *complicate assessment *are the subjective nature of reflection's content and the dependency on descriptions by persons being assessed about their reflection process, without any objective means of verification. To counter these validity threats, we suggest that assessment should focus on generic process skills rather than the subjective content of reflection and where possible to consider objective information about the triggering situation to verify described reflections. Finally, *internal and external contextual factors *such as motivation, instruction, character of assessment (formative or summative) and the ability of individual learning environments to stimulate reflection should be considered.

## Background

Physicians and other healthcare workers act in challenging professional environments. There is an exponential growth in knowledge and treatment options, patients are becoming more articulate and demanding, and inter-professional collaboration is the rule rather than the exception. Lifelong learning is, consequently, crucial to the provision of up-to-date healthcare services [1]. Rather than just attending conferences, lifelong learning today is seen as a continuous process, embedded in everyday professional practice. At its core lies practitioners' ability to reflect upon their own actions, continuously reviewing the processes and outcomes of treatments, defining new personal learning objectives, and planning future actions in pursuit of excellence [2-5]. Hence, the ability to reflect is an important outcome parameter for health care professionals [6-9]. As a result, many educational institutions incorporate the ability to reflect as an objective of their vocational programs, premised on a belief that reflective thinking is something that can be developed rather than a stable personality trait [4,10,11].

There is, however, uncertainty about how best to help people develop their ability to reflect [11]. Lack of an agreed way of assessing reflection is a particular obstacle to progress because assessment is needed for the identification of effectiveness of educational strategies and for research purposes [3]. Assessment has also a motivational influence as a source for feedback (formative assessment) and when to judge whether requisite levels of competence have been attained (summative assessment) [3,4,12]. The persisting lack of clarity about how to operationalise reflective learning is symptomatic of an even deeper problem. Different, widely accepted theories define reflection in different ways, consider different outcomes as important, define different dimensions along which reflection could be assessed and point towards different standards [11]. Consequently, research findings are hard to compare. This unsatisfactory state of affairs leaves curriculum leaders without practical guidelines, ways of identifying and supporting students who are weak reflectors, and ways of judging whether interventions are improving learners' ability to reflect. The purpose of this article is to review four factors, which confound the assessment of reflection:

1. Non-uniformity in defining reflection and linking theory with practice.

2. A lack of agreed standards to interpret the results of assessments.

3. Threats to the validity of current methods of assessing reflection.

4. The influence of internal and external contextual factors on the assessment of reflection.

Our approach was to identify all widely quoted theories, read them in depth, and triangulate them against one another to find what they (dis)agreed on and gaps between them. The result of this exercise was an interpretive framework, which we used to structure the 'Discussion' section. A test of the framework is beyond the scope of this article, whose aim is to make the framework and guidelines available to other people interested in implementing and/or assessing reflection in education.

## Discussion

### 1. Defining reflection

Studies about reflection in professional practice and education are widespread in the literature; however, their results are hard to generalise or compare because they conceptualise reflection in such different ways. Boenink et al [10] described reflection in terms of the number of different perspectives a person used to analyse a situation. Reflection ranged from a single perspective to a balanced approach considering multiple relevant perspectives. Aukes et al [13] emphasised emotional and communication components when they conceptualised personal reflection as a combination of self-reflection, empathic reflection, and reflective communication. Sobral's [14] emphasis on reflection-in-learning approached reflection from a learning perspective.

If those three perspectives exemplify inconsistency in the field, the work of Dewey, Boud, Schön, Kolb, Moon, and Mezirow exemplifies shared ground between reflection theories and used terms. *Dewey *is usually regarded as the founder of the concept of reflection in an educational context. He described reflective thought as "active, persistent, and careful consideration of any belief or supposed form of knowledge in the light of the grounds that support it, and the further conclusions to which it tends" [15]. He saw **reflective thinking **in the education of individuals as a lever for the development of a wider democratic society.

In line with his work, *Boud et al *emphasised reflection as a tool to learn from experience in **experiential learning **[16]. They identified reflection as a process that looks back on experience to obtain new perspectives and inform future behaviour. A special feature of their description of reflection in three stages - 1. Returning to an experience; 2. attending to feelings; and 3. re-evaluating the experience - was the emphasis it placed on the role of emotions.

*Moon *described reflection as an input-outcome process [17]. She identified reflection as a mental function transforming factual or theoretical, verbal or non-verbal knowledge, and emotional components generated in the past or present into the output of reflection (e.g. learning, critical review or self-development).

*Schön's *concept of the **reflective practitioner **identified reflection as a tool to deal with complex professional situations [18,19]. Reflection in a situation (reflection-in-action) is linked to practitioners' immediate behaviour. Reflection after the event (reflection-on-action) provides insights that can improve future practice. Those two types of reflection together form a continuum for practice improvement.

The term **'reflective learning' **describes reflection in the context of experiential learning. *Kolb's *widely accepted experiential learning cycle describes four stages of learning: 1. having an experience (concrete experience), 2. reflective observation (reflecting on this experience), 3. abstract conceptualisation (learning from the experience) and 4. active experimentation (trying out what you have learned) [20]. These four stages are conceptualised as a spiral, each of whose turns is a step forward in a person's experiential learning.

**Lifelong learning **is considered today as essential for maintaining a high standard of professional practice. *Mezirow's *transformative learning theory described lifelong learning in terms of learners' transforming frames of reference, in which reflection is the driving force [21].

#### Towards an 'eclectic model' of common elements

Although contemporary reflection models build on those theories, the diversity between them is a cause of continuing uncertainty. In response, we have assembled a simple comprehensive model from their common parts (table 1). Atkins and Murphy [22] identified reflection as: 1. 'awareness of uncomfortable feelings and thoughts', resulting in 2. an 'analysis of feelings and knowledge', finally leading to 3. 'new perspectives'. They described self-awareness, critical analysis, synthesis, and evaluation as requisite skills for this process. Since those three phases are common to the work of previous authors, they provided a logical starting point for our model. We complemented Atkins and Murphy's phases with insights from other models. Korthagen's ALACT model ('Action, Looking back on action, Awareness of essential aspects, Creating alternative methods of action, and Trial') [23] describes the first phase of 'becoming aware' in two steps: a general retrospective action and a more interpretive action. Integrating those two theories, resulted in a first phase ('reviewing an experience') with two subcomponents: 1. generally describing what happened and 2. identifying essential aspects by considering both thoughts, feelings and contextual factors.

**Table 1:** Overview of theories/models/findings integrated into the model of common elements

Author	Theory/Model/Finding	Summary
Atkins & Murphy	Reflective processes (model)	Three key stages of the reflective process: 1. Awareness of uncomfortable feelings, 2. Critical analysis of feelings and knowledge, 3. New perspectives; associated skills: self-awareness, description, critical analysis, synthesis and evaluation.
Boud	Promoting reflection in learning (model)	Reflection process consists of 3 interrelated stages: 1. returning to the experience, 2. attending to feelings, 3. re-evaluating experience, triggered by experiences and leading to outcome.
Bourner		Interrogating experiences with searching questions distinguishes reflective from unreflective thinking.
Korthagen	ALACT model	Reflection is a cyclic process of: Action, Looking back on action, Awareness of essential aspects, Creating alternative methods of action, and Trial.
Mamede & Schmidt	The structure of reflective practice in medicine (finding)	Reflective practice consists of a 5 factor model: deliberate induction; deliberate deduction; testing and synthesising; openness for reflection, and meta-reasoning.
Mezirow	Transformative learning theory	Reflection leads to changed assumptions and frames of references which ground transformative learning.
Moon	Input-outcome model	Reflection is a mental process that is based on input (theories, constructed knowledge or feelings) that has an outcome/purpose (self-development, learning, decisions, resolutions of uncertainty, ...).
Schön	The reflective practitioner (theory)	Reflection is a key factor for professionals to deal with complex situations and for professional development. He identified reflection-in-action and reflection-on-action.
Stockhausen	Clinical learning spiral (model)	Reflective practice is related to professional growth; Clinical learning consists of preparative phase, constructive phase, reflective practice and reconstructive phase.

Just reviewing an experience, however, does not necessarily lead to effective reflection. For Bourner [24], using searching questions to interrogate an experience was the key difference between reflecting and thinking and he saw 'reflective inquiry' as a crucial component of reflection. This aspect of reflection was also represented in Mamede and Schmidt's proposed structure of reflective practice as 'openness to reflection' [25]. Bourner only emphasised posing searching questions, however, not answering them. Korthagen's approach supplements Bourner's by contributing 'creating alternative methods of action' as a process of answering questions. This addition is compatible with Boud's characterization of analysis as a combination of association, integration, validation and appropriation. The internal dialogue that results is conducted within a 'personal frame of reference' that, according to Mezirow, directs the analysis and represents "the structure of assumptions and expectations through which we filter sense impressions" [21]. This personal perspective, made up of our perceptions, cognitions, feelings and dispositions (intentions, expectations and purposes), creates a context in which we give meaning to our sensory experiences. If the first phase of reflection, then, is identified as the description of an experience and the awareness of feelings, thoughts, and other essential aspects, our second phase of reflection is analysing experiences by reflective inquiry, which triggers a process of analysis within a person's unique frame of reference.

Moon's input-outcome model emphasises that reflection is purposeful [17]. This purpose is identified by Atkins and Murphy in the third phase of reflection as the 'identification of new perspectives' [22]. Both Korthagen and Boud, however, included an additional stage - the conversion of those new perspectives into actions that are the starting point for new reflective cycles [16,23]. The 'reconstruction phase' of Stockhausen's clinical learning spiral model of reflective practice among undergraduate nursing students in clinical practice settings had the same function [26]. During this phase, reflective insights were transformed into plans for future actions. Since those actions could lead to further reflections, reflecting on experiences was identified as a cyclic process that transformed significant experiences into deliberate, well informed practical actions. We incorporated those insights into the eclectic model by defining the outcome of a reflection process as the identification of new perspectives, which leads to future actions informed by reflection. Stockhausen also described a preparatory phase to establish objectives for a new clinical experience. This phase, which other authors have labelled as reflection-before-action [27,28], is incorporated into the eclectic model by representing reflection as a cyclical process. It allows reflection to be informed by learning goals arising from past reflections and stresses the importance of reflection as a developmental process. Both Korthagen and Stockhausen have highlighted this process with the term reflection spiral with each winding leading to a higher order of understanding, practice or learning [23,26].

**Figure 1:**
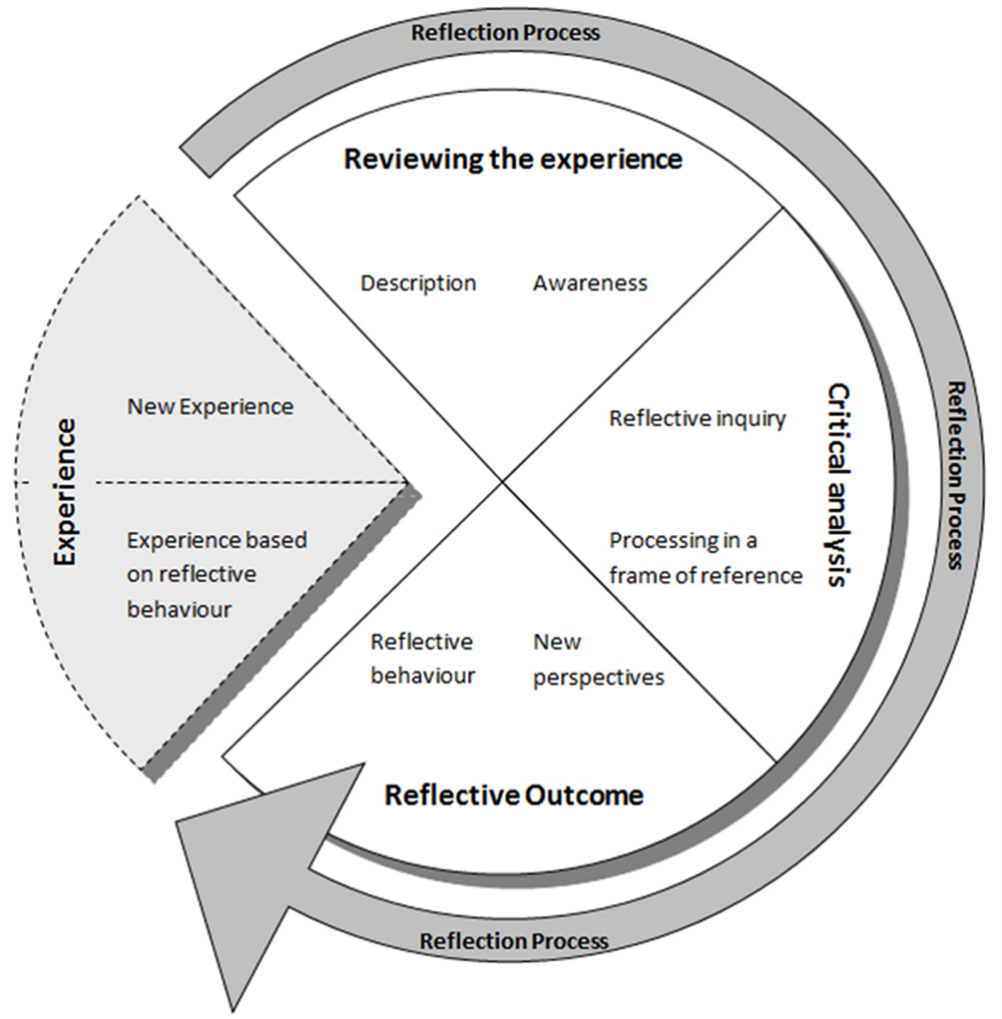
**Model of common elements describing the reflection process**.

Figure 1 shows the complete eclectic model, which describes reflection in three phases: 1. 'Reviewing the experience', 2. 'Critical analysis', and 3. 'Reflective outcome'. Reflection, according to the model, is a cyclical process, which originates from experience and informs future behaviour. Each phase has two items, described in practical terms to make it possible to put the model into practice. Reviewing the experience has two components: 'description of the experience as a whole', and 'awareness of essential aspects based on the consideration of personal thoughts, feelings, and important contextual factors'. Critical analysis starts with 'reflective inquiry' - posing searching questions about an experience - and progresses to 'searching for answers' while remaining aware of the 'frame of reference' within which the inquiry is being conducted. Reflective outcome comprises the 'new perspectives' resulting from phase two, and the 'translation of those perspectives into behaviour that has been informed by reflection'. This behaviour generates new experiences and so a new reflection cycle begins.

#### From model building to developing indicators for assessment of reflection

The aim of identifying common elements was to ground the assessment of reflection in existing, widely used theories. It is practically useful because each of the six items in the three phase model can be translated into an indicator of the adequacy of reflection processes (table 2). Together, they provide a comprehensive overview of a person's ability to go through the process and are in line with the reflective skills identified by Duke and Appleton [29]. Taken individually, the indicators can provide specific feedback about components of reflection, which makes it possible to give structured, focused feedback, and direct training towards aspects of reflection that the indicators have defined as insufficient. Such training could, for example, provide exercises on describing personal thoughts and feelings or identifying learning goals. So, in summary, the modular nature of the model and its indicators makes it possible to tailor education to individual needs. But, for that, criteria to judge someone as competent in reflection are needed.

**Table 2:** Operational indicators of the reflection process

Aspect of the reflection process	Indicators	
Reviewing the experience	1.	The ability to describe an event/situation adequately.
	2.	The ability to identify essential elements and to describe own thoughts and feelings
Critical analysis	3.	The ability to ask searching questions.
	4.	The ability to answer searching questions and being aware of the frames of references in use.
Reflective outcome	5.	The ability to draw conclusions.
	6.	The ability to describe concrete learning goals and plans for future action.

### 2. Standards to interpret reflection assessment

Here, again, there is a lack of consensus in published literature. A few researchers have attempted to rank reflections. Wong et al [30] evaluated reflection in written papers by identifying reflective activities using two coding schemes. One, based on Boud's theory, had six items: attending to feelings, association, integration, validation, appropriation and outcome of reflection. The other, based on the work of Mezirow, labelled students as: non-reflectors (no evidence of reflective thinking), reflectors (evidence of relating experience to learning opportunities) and critical reflectors (evidence of integrating reflective outcomes in professional behaviour). The researchers found Boud's categories hard to apply to written materials, resulting in less reliable coding than using Mezirow's scheme. With only three categories, however, this latter scheme had a limited capacity to discriminate between people. Kember et al [31] addressed this issue by using a finer-tuned coding scheme based on the work of Mezirow. Their seven categories ranged from unreflective thinking (habitual action, introspection and thoughtful action) to reflective thinking (content reflection, process reflection, content and process reflection and premise reflection). They dealt with the complexity of the coding scheme by providing guidelines for assessors, which resulted in an acceptable interrater reliability (Cronbach alpha 0.74). Boenink et al [10] used an alternative approach, which ranked reflections from 1-10. Their scale was based on the number of perspectives students described in short written reflective reactions to a case vignette describing a challenging situation. The advantage of this approach was the limited need to make interpretations when identifying the perspectives. The scale was limited, however, by measuring only one aspect of reflection (being aware of the frame of reference used). Duke and Appleton [29] developed a broader marking grid to score reflective reports. It assessed eight skills that support reflection, identified by a literature review, on five-level scales, 'anchored' and linked to a grade (A, B^+^, B, C and F). By providing grades, these authors were the first to set standards for reflective skills. Despite having based the reflection skills that were included in the scale on a literature review, however, the authors did not disclose how they linked the levels to grades.

Coding schemes have also been used to evaluate reflection in interviews. Boyd [32] assessed reflective judgement using a coding scheme based on seven stages of intellectual development described by King and Kitchener: Pre-reflective thinking (stages 1-3); quasi-reflective thinking (stages 4 and 5); and reflective thinking (stages 6 and 7). Measurements made with the scale had an interrater reliability of 0.76 (Cronbach alpha).

Based on the approach coding schemes can be divided into two groups. A first approach ranks reflections according to levels, ranging from descriptive and/or unreflective to reflective or critical reflective based on the used theory [30-32]. The other approach is the identification of phases in the reflection process considering items of reviewing an experience, analysis and reflective outcome based on the used model of reflection [29,30]. This discrepancy is a complicating factor for interpreting results as levels and phases are incompatible.

Notwithstanding limited ability to compare the findings in the reported studies, because of the variety in used the scales and models of reflection, their results share a common feature. Within their own scale, all studies demonstrate learners to have very limited mastery of reflection, indicating an apparent room for improvement. Inadequate reflection has a negative effect on practice [3,18,33], presumably because learners with a limited ability to reflect let 'tunnel vision' stop them questioning their behaviour in response to significant positive and negative experiences [18,34]. That situation need not be left unchallenged because there is research showing reflection can be influenced positively by training [14,32,35], but the minimum level of reflection needed to have a positive effect on practice remains to be defined.

Until standards have been formulated that can identify practitioners whose level of reflection is adequate, it seems reasonable to clarify to stakeholders (curriculum developers, students, practitioners, assessors) what reflection skills are expected and urge learners to develop them as far as possible. We offer the presented model of common elements as a way of doing that. In promoting reflective learning, however, a balance has to be struck between developing an ability to reflect and increasing the frequency of reflection. It has been argued that critically analysing personal practice after every experience can cause a disabling level of uncertainty [36,37]. Future standards will therefore have to consider the balance between the quality of reflection and its efficient and systematic application in practice, but not to the stage of being counterproductive.

### 3. Factors that complicate assessment

The metacognitive nature of reflection is an important complicating factor of reflection assessment [4]. It implies a thinking process only accessible to the reflecting person and hence only observable by assessors through that person's interpretative descriptions. Subjects are most often asked to 'translate' their reflections into written words, which are assessed against coding schemes or scoring grids [29-31,38-40]. Other suggested methods to 'visualise' reflections include the verbalisation in interviews [32,41,42], written responses to vignettes [10], or reflective writings in portfolios [34,43]. Assessors' dependency on a person's interpretative description is a serious threat to the validity of assessments of reflection because they have to judge selective descriptions without being able to verify their adequacy. Accordingly this approach fails to detect bias caused by a lack of (un)intentional hindsight and introspection ability [44,45], reflections being determined by the requirements of the assessment and selectivity and/or incompleteness of aspects they portray [44]. Interviews have the advantage that they can pose clarifying questions and monitor a reflecting person's reactions, but they still leave assessors to ground their judgements in potentially subjective and selective narrative accounts of reflective activity. There are two related problems in that. Although the semantic skill of describing reflections is considered integral to effective reflection [46], skills other than pure reflective skills are needed to turn reflection into writing and/or speech, which has a self-evident effect on reflective narratives [44]. The other problem lies in a decrease of motivation caused by the non-alignment between the written approach to assessment and a learners preferred learning style [12]. Findings of Sandars and Homer [47] suggest the discrepancy between 'net generation' students learning preference of group-based and technological multimedia activities (blogs, social networks, digital storytelling) and the text based approaches to reflective learning. Moreover, supporting learners to reflect with the creative use of multimedia, will likely increase their commitment to reflect and stimulate even more efficient reflection [48].

Self-assessment questionnaires have the advantage of circumventing indirect observation [13,14,49,50], but their requirement to introspect accurately introduces another validity threat [22,51], because it is then unclear if it is reflection or the ability to introspect that is being tested. Eva and Regehr [45] concluded that it is best not to build solely on self-assessment approaches as they tend to be inaccurate and they recommended triangulating introspection with other forms of feedback. Assessor-based methods could meet this requirement, providing assessors could be relied upon to provide valid feedback.

Since there are such serious validity threats, the question remains whether it is possible to assess reflection at all. Two elements appear to be important. In search for a valid approach, Bourner [24] suggested the content and the process of reflection should be viewed as two separate entities. While the content is a barrier to assessment because of its subjective nature, the process has a more general character. He transferred this approach from the assessment of critical thinking where the use of questions to analyse ideas, evidence and assertions demonstrates a person's capacity for critical thinking [24]. Similarly Bourner proposed that observable items, like the ability to formulate learning goals, should be used to demonstrate a person's capacity for reflecting. This approach demonstrates some parallels with the content specificity of clinical reasoning [52]. However, opposed to elements in reflections such as learning goals or plans for future actions which meaning for the learner is subjective, content specific knowledge has a more objective character.

Furthermore, reflections are intimately linked to their triggering situations [16,18,19,53] so information about this initial event can provide an objective frame of reference to verify elements of the reflection. For example, when someone describes his communication as good, the real-time presence of an assessor or video-recording of the event could give supporting information [54]. Finding a feasible way of obtaining a rich picture of events that precede the reflection that has to be assessed is an important topic for future development.

### 4. Internal and external contextual factors affecting reflection assessment

The results of assessments of reflection are influenced by contextual factors as well as people's ability to reflect. Our argument now turns to those modulating factors. Motivation is considered to be an important mediator of learning and achievement in medical education [55,56]. The expectancy-value model proposed by Wigfield and Eccles identifies the subjective value of a task to a person and their expectation of performing it successfully as main predictors of task performance [57]. Applied to reflection, it predicts that the perceived importance of reflection for (professional) practice will determine the time and effort a person is willing to invest in it; those who do not expect a positive return are unlikely to reflect profoundly and critically [4]. This motivational model also explains how personal factors like prior experience of reflective learning and a person's understanding of the reflection process will influence motivation and consequently reflective behaviour. Hence introductory sessions are important to frame the value and intended outcomes of reflection [4]. Furthermore the expectancy-value model also stresses external variables, which might include aspects of teaching and/or assessment. It is reciprocal in nature. If involvement in reflective activities results in perceived better performance (internal) and/or external appraisal, rewards, or reinforcement, a feedback loop starts to operate.

Whereas reflection was traditionally conceived of as a strictly individual process, ideas are shifting towards conceptualising it as a process facilitated by social interaction [4,45]. A stimulating environment in which supervisors and peers give learners regular feedback and ask thought-provoking questions can, from that point of view, be expected to improve reflection. With non-judgemental questions, facilitators can encourage to fully explore the situation, to consider alternative perspectives and solutions, and to uncover taken-for-granted assumptions [3]. Furthermore, situations and reflection upon can provoke strong emotions and negative thoughts which could potentially form a barrier obstructing efficient reflection. A facilitator can help to assimilate these strong emotions and refocus on the reflection process [12,16]. To fully explore reflective thoughts, feelings and possible emotions, it is crucial to create a safe environment established between the reflecting person and the facilitator(s) [3]. Next to supporting others, being a facilitator is also reported as even more effective for a person's own reflections [58]. Schön, however, warned that an unbalanced relationship between learner and coach and an undue influence of contextual factors could hinder reflective practice, as it could lead to defensiveness [18]. In line with this emphasis on contextual factors, Schaub et al developed a scale to assess teachers' competence in encouraging reflective learning [59]. It asks learners to identify whether teachers support self-insights, create a safe environment, and encourage self-regulation.

Because of their influence on reflections contextual factors should be accounted for in educational and assessment approaches. In education it will help to develop effective educational strategies and predict their results to match the intended outcome. In assessment considering contextual factors will contribute to the interpretation of results and in the understanding of the reflection process. Hence we suggest to consider internal and external contextual factors in education and assessment.

## Summary

Whilst it is generally accepted that the ability to reflect is an important attribute for healthcare professionals, there is considerable uncertainty about how best to foster it in educational practice. Lack of an agreed way of assessing reflection is a very important factor contributing to this uncertainty. There is, however, clearly discernible common ground between reflection theories. By defining that common ground, we have been able to assemble an eclectic model, which sees reflection as comprised of: 1. reviewing experience; 2. critical analysis; and 3. reflective outcomes. A way of reliably measuring reflection is needed so summative judgements can be made and learners can receive effective feedback but one has not, yet, been developed. Standards defining an essential minimum level of reflective ability are also needed. Until they are we urge to develop and communicate a local consensus between stakeholders (students, practitioners, teachers, supervisors, curriculum developers) about what is expected in exercises and formal tests.

Because reflection is a metacognitive process, it can only be assessed indirectly; through written reflections in vignettes or portfolios, or spoken expressions in interviews. These methods do not allow assessors to verify information related to the reflections reported, which is a serious limitation. The widespread use of self-assessment questionnaires shares both that validity problem and the inherent limitations of self-assessment. To counter these validity threats, it has been proposed that assessment should focus on the process rather than the subjectively coloured content of reflection. In addition, as reflections are intimately entangled with their triggering situational context, we suggest where possible to consider objective information about this triggering situation allowing assessors to verify described reflections. The reflection process is influenced by internal (eg. motivation, expectancy and prior experiences with reflection) and external factors (formative or summative character of assessment, presence of facilitators and introduction to the assessment). Awareness of these factors are important to develop effective educational strategies, interpreting assessment results and finally the increase in understanding about the reflection process. Based on the preceding discussion, we offer the following practical guidelines for educating and assessing reflection.

1. Clearly define the concept of reflection and verify that all stakeholders (curriculum developers, students, assessors and supervisors) adopt the same definition and intended outcomes.

2. Be specific about what level of reflection skills is expected, identifying good and inadequate reflection and communicate this to all stakeholders.

3. Be aware of possible bias in self-assessment methods, caused by inadequate ability to introspect.

4. Provide assessors with a perspective on the situation triggering the reflection to create the ability to verify the described reflections in an objective frame of additional information.

5. Consider and report contextual factors when assessing reflection and/or when engaging in reflective education in support to interpret the outcomes.

## Competing interests

The authors declare that they have no competing interests.

## Authors' contributions

SK conceptualized the idea and SK, TD were involved in writing the initial drafts. All authors were involved in the revised drafts and made essential contributions to this paper and critically reviewed and approved the final manuscript.

## Pre-publication history

The pre-publication history for this paper can be accessed here:

http://www.biomedcentral.com/1472-6920/11/104/prepub
